# Sex Differences in the Allele Distribution of *PGLYRP2* Variant rs892145 in Parkinson's Disease

**DOI:** 10.1155/2023/6502727

**Published:** 2023-12-09

**Authors:** Caroline Ran, Karin Wirdefeldt, Olof Sydow, Per Svenningsson, Rochellys Diaz Heijtz

**Affiliations:** ^1^Department of Neuroscience, Karolinska Institutet, Solna, Sweden; ^2^Department of Clinical Neuroscience, Karolinska Institutet, Solna, Sweden; ^3^Department of Medical Epidemiology and Biostatistics, Karolinska Institutet, Solna, Sweden

## Abstract

**Introduction:**

Parkinson's disease (PD) is a complex multifactorial disease, involving genetic susceptibility, environmental risk factors, and gene-environmental interactions. The microbiota-gut-brain axis is hypothesized to play a role in the pathophysiology of PD, and peptidoglycan recognition proteins (PGLYRPs), which modulate the gut microbiota, are, therefore, relevant candidate genes for PD.

**Methods:**

Using quantitative real-time PCR, we genotyped three *PGLYRP* variants (rs892145, rs959117, and rs10888557) and performed an association analysis in 508 PD patients and 585 control individuals. We further conducted a meta-analysis of rs892145 and analyzed *PGLYRP2* gene expression in lymphocytes from patients with PD and controls.

**Results:**

Although initial analysis of the three variants rs892145, rs959117, and rs10888557 and a meta-analysis of rs892145 did not reveal any association between the selected variants and PD, we found an interaction between sex and genotype for rs892145, with a marked difference in the allele distribution of rs892145 between male and female patients. As compared to controls, the T allele was less common in female patients (odds ratio = 0.76, *P* = 0.04) and more common in male patients (odds ratio = 1.29, *P* = 0.04). No difference was found in *PGLYRP2* gene expression between PD patients and controls (*P* = 0.38), nor between sexes (*P* = 0.07). *Discussion*. Overall, this genetic screening in Swedish PD patients does not support previous results demonstrating associations of *PGLYRP* variants with the risk of PD. Meta-analysis of rs892145 revealed pronounced heterogeneity between previously published studies which is likely to have influenced the results. Taken together, the genetic and gene expression analyses suggest a possible link between genetic variants in *PGLYRP2* and sex differences in PD. Because of the limited sample size in our study, these results need to be verified in independent cohorts before concluding.

## 1. Introduction

Parkinson's disease (PD) is a common neurodegenerative disorder affecting 1% of the population aged over 65. PD is a devastating disorder which is recognized by the pronounced degeneration of dopaminergic neurons of the substantia nigra, though other neurons are also affected [1, 2]. A hallmark of PD pathology is the presence of Lewy bodies (LBs), neuronal inclusions consisting of *α*-synuclein, and other proteins [3, 4]. It is not known how the pathology of PD is initiated in the affected brain. One hypothesis is that PD originates in the gut, which is supported by frequent descriptions of gastrointestinal dysfunction that occurs before the onset of motor symptoms and clinical diagnosis of PD. Furthermore, LBs have been described in the enteric nervous system of patients with PD as well as in prodromal patients, several years before diagnosis [5, 6]. The notion of PD originating elsewhere than in the affected areas of the brain is supported by the Braak stages, describing neuropathological findings progressing from the lower brainstem, or periphery, towards higher cortical regions in the final stages of the disease [7, 8]. In addition, *α*-synuclein pathology and gastrointestinal dysfunction have been observed prior to the onset of motor symptoms in a transgenic mouse model of PD expressing a mutated form (A53T) of human *α*-synuclein [9].

Accumulating evidence suggests that the microbiota is affected in PD, as in several other neurological disorders. Studies have shown that proinflammatory bacteria are more abundant in the fecal microbiota of PD patients than those in healthy control individuals [10] and that the shift in bacterial species may be linked to the severity of certain motor symptoms [11]. These interactions have been confirmed in *α*-synuclein overexpressing mice. Microbiota transplanted to these mice from PD patients were discovered to worsen the parkinsonian phenotype, while germ-free *α*-synuclein overexpressing mice displayed alleviated motor symptoms as well as reduced *α*-synuclein pathology in the brain [12].

Peptidoglycan recognition proteins (PGLYRPs) are known to modulate the innate immune response in order to maintain a healthy gut microbiota, including eliminating harmful bacteria [13]. PGLYRPs are membrane proteins recognizing peptidoglycan proteins, which are important constituents of the bacterial membrane. Humans have four PGLYRPs, three of which have previously been suggested to be involved in PD. In 2014, Goldman et al. reported genetic associations with rs892145 in *PGLYRP2*, rs2987763 in *PGLYRP3*, and several variants in *PGLYRP4*, of which the strongest association was observed for rs10888557 [14]. Genetic variants in *PGLYRP2* and *PGLYRP4* were then replicated as genetic risk factors for PD in an Australian cohort, and variants in *PGLYRP2* were replicated in a Chinese cohort [15, 16]. Although these variants have not previously been suggested as PD risk factors in GWAS analysis, we suggest they are worth investigating in independent well-characterized cohorts as the genetic interactions are possibly influenced by environmental factors linked to the microbiota.

In an attempt to validate the *PGLYRP2* and *PGLYRP4* genes as candidate genes for PD, we investigated genetic variants in *PGLYRP2* (rs892145) and *PGLYRP4* (rs10888557) in a Swedish PD cohort. As the *PGLYRP2* variant rs892145 was a nonsynonymous variant, we used the TagSNP selection tool (SNPinfo) to find additional single nucleotide polymorphisms (SNPs) for genotyping in *PGLYRP2* [17]. Of the three suggested tag-SNPs, rs959117 was in very low linkage disequilibrium (LD) with rs892145 and therefore selected to cover more variants in the gene. We also performed a gene expression analysis, comparing the expression of our lead candidate gene *PGLYRP2* in PD patients and healthy control individuals.

## 2. Methods

### 2.1. Material

We analyzed DNA samples from 508 patients with a diagnosis of idiopathic PD, according to the UK Parkinson's Disease Society Brain Bank criteria for idiopathic PD without applying the exclusion criterion of having a close relative with PD, and from 585 control subjects [18]. PD patients were recruited at the neurology clinic at the Karolinska University Hospital. The control population consisted of 286 individuals from the SNAC-K project (the Swedish National Study on Aging and Care in Kungsholmen, https://www.snac-k.se/), 190 anonymous blood donors, and 109 neurologically healthy individuals from the Stockholm area also recruited at the Karolinska University Hospital. The demographics of the study population are shown in Table 1. The experiments described in this report were performed according to the Declaration of Helsinki and after obtaining approval from the Ethical Review Board of Sweden. After giving their informed consent, participants provided a blood sample from which DNA was extracted. They also provided relevant clinical information.

Gene expression was analyzed in Epstein–Barr virus (EBV) transfected B lymphocytes from 20 individuals. The procedure of EBV transfection has been described elsewhere [19]. Frozen cell pellets were then used for RNA extraction (RNeasy Mini Prep Kit, QIAGEN Nordic, Sollentuna, Sweden) and cDNA synthesis (Quantitect Reverse Transcription Kit, QIAGEN) according to the manufacturer's instructions.

### 2.2. Genotyping

Genetic variants were selected from the literature and using SNPinfo [14, 17]. Genotyping was carried out with quantitative real-time PCR (qPCR) on an ABI 7500 Fast system (Applied Biosystems, Foster City, CA, USA). We used premade TaqMan® assays for all three SNPs (C___7494113_10 for rs892145, C___8157038_10 for rs959117, and C__31623108_10 for rs10888557) and TaqMan® Genotyping Master Mix for the PCR reaction (Thermo Fisher Scientific Inc., Fisher Scientific, Gothenburg, Sweden). The cycler was programmed to perform 50 cycles of denaturation at 95°C for 15 seconds and annealing/extension at 60°C for 1 minute. Genotypes were determined using the 7500 Software v2.0.6 supplied with the TaqMan instrument (Applied Biosystems). SNPs rs892145 and rs959117 were genotyped in 1093 individuals with a call rate of 98% and 96%, respectively. rs10888557 was genotyped in 846 individuals with a call rate of 98%.

### 2.3. Gene Expression

Gene expression was assessed in 13 PD patients and seven controls by means of reverse-transcription qPCR (RT-qPCR). We used a CFX96 cycler (Bio-Rad Laboratories AB, Solna, Sweden) programmed to run 40 cycles of denaturation at 95°C for 5 seconds and annealing/extension at 60°C for 30 seconds. We used Bio-Rad Prime-PCR™ SYBR® green probes for target gene *PGLYRP2*(qHSACED0045927) and reference genes TATA-box binding protein (*TBP*) (qHsaCID0007122) and pyruvate dehydrogenase E1 subunit beta (*PDHB*) (qHsaCED0046539) combined with the SsoAdvanced™ Universal Probes Supermix (Bio-Rad).

### 2.4. Statistical Analysis

Association analysis, Hardy–Weinberg Equilibrium (HWE) analysis, and meta-analysis were performed in PLINK v1.9 [20]. Allele association with PD was investigated using logistic regression with an additive model and sex as a covariate, as PD is more common in men (62.8% males in our patient group, Table 1). We used a formal interaction test to test for an interaction between rs892145 and sex and further used the Breslow–Day test to verify our findings. Sex-stratified analysis included the 1090 individuals for which we had information on sex.

We performed a meta-analysis on rs892145, including our own data and data from three other studies [14–16]. The Australian study was analyzed as two separate cohorts and was, therefore, included as two datasets here [15]. In all three previously published studies, the heterozygous vs. wild-type genotype of rs892145 was analyzed and deemed the most significant model. For conformity between cohorts in the meta-analysis, we tested the association of said model for rs892145 also in the Swedish material using a chi-squared (*X*^2^) test in GraphPad Prism 5.03 (GraphPad Software Inc., La Jolla, CA, USA). We ran the meta-analysis using a random effects model in which 95% confidence intervals were computed and a forest plot was generated using R v.4.1.2.

Gene expression data were analyzed in CFX Manager v3.1 software (Bio-Rad) and GraphPad Prism 5.03 (GraphPad Software Inc.). mRNA levels were normalized to reference gene *TBP* (*PDHB* had to be excluded from the analysis due to high variability in replicates) and to a random reference sample. Data were log-transformed and analyzed with a student's *t*-test and two-tailed *P* values. *P* values lower than 0.05 were considered significant.

## 3. Results

We performed a candidate gene study investigating three genetic variants in the *PGLYRP2* and *PGLYRP4* genes in relation to PD. rs892145 and rs959117 in *PGLYRP2*, and rs10888557 in *PGLYRP4* did not deviate from HWE in the control or patient groups, except for rs959117 which deviated slightly in cases, *P* = 0.047. Statistical analysis performed with logistic regression using sex as a covariate showed that there was no significant difference in allele frequencies between cases and controls for any of the three variants (Table 2). We further ran a formal interaction test, investigating the interaction between genotype and sex, which gave a significant association for rs892145 with a *P* value of 0.009. As a consequence, we stratified the material and analyzed male and female subjects separately (Table 2). Interestingly, the allelic distribution of the *PGLYRP2* rs892145 variant was found to vary between males and females. In female PD patients the minor allele T was less common than in controls (odds ratio (OR) 0.76, *P* = 0.04). The opposite relation was true in males, with an overrepresentation of the T allele in patients (OR 1.29, *P* = 0.04). The Breslow–Day test confirmed that the ORs in male and female strata were significantly different (*X*^2^ = 8.41, *P* = 0.004) and should, therefore, be analyzed separately.

**Figure 1:**
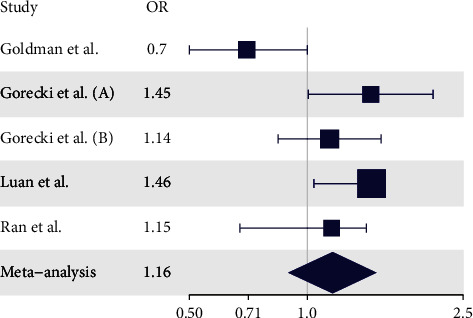
Forest plot from the rs892145 meta-analysis. Meta-analysis of four studies analyzing the association of rs892145 in *PGLYRP2* and PD. The analysis was run under a random effect model because of elevated heterogeneity between studies, Cochrane's *Q* statistic *P* value 0.0041, *I*^2^ heterogeneity index 73.83.

Last, we performed a meta-analysis including four studies analyzing the association of rs892145 (including our own) (Figure 1) [14–16]. The Cochrane's *Q* test for heterogeneity was highly significant (*P* value = 0.0041, *I*^2^ heterogeneity index = 73.83), and we, therefore, analyzed the association under a random effects model. The meta-analysis gave an overall OR of 1.16, *P* value 0.23, with a confidence interval overlapping zero.

**Figure 2:**
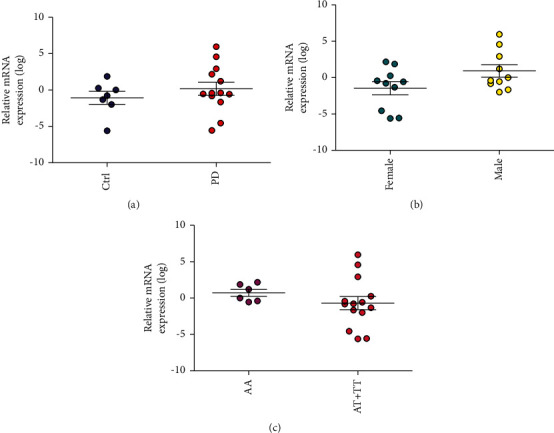
*PGLYRP2* expression levels in B lymphocytes. *PGLYRP2* mRNA levels in PD patients (*n* = 13) and controls (*n* = 7). Data were normalized to housekeeping gene *TBP* and to a control reference sample. Data were log-transformed, and expression levels were analyzed with a student's *t*-test, two-tailed *P* value, with respect to (a) diagnosis (*P* = 0.38), (b) sex (*P* = 0.07), and (c) rs892145 allele (*P* = 0.35). C: Control, PD: Parkinson's disease, AA: individuals carrying two copies of the major allele, and AT + TT: individuals carrying one or two copies of the minor (T) allele.

Results from the gene expression experiments showed relatively low *PGLYRP2* expression in B lymphocytes with an average cycle threshold (Ct) of 30.6. The analysis did not indicate any difference in relative mRNA levels of *PGLYRP2* between PD patients and controls (Figure 2(a), *P* value = 0.38). Given the results from our genetic analysis, we compared gene expression in males and females regardless of PD status. Our analysis revealed a trend for lower *PGLYRP2* expression in females (Figure 2(b), *P* value = 0.07). Because of a low number of homozygous TT carriers for rs892145, we compared individuals with one or two copies of the minor (T) allele to homozygous AA carriers. The presence of the minor allele of rs892145 did not affect the overall *PGLYRP2* expression (Figure 2(c), *P* value = 0.35).

## 4. Discussion

In this study, we investigated three SNPs in *PGLYRP2* and *PGLYRP4* that have previously been suggested as risk factors for PD [14–16]. Our association analysis did not confirm any association between these genetic variants and PD. Furthermore, our gene expression analysis, comparing the relative mRNA levels between PD patients and controls, revealed comparable *PGLYRP2* gene expression in the two groups. The discrepancy between our results and those reported previously could have several explanations. The studies used different methodologies and models of analysis. Although three studies (Goldman et al., Gorecki et al., and the present study) were based on Caucasian populations, there is also some difference in population background (American/Australian/Swedish) [14, 15]. Associations identified between PD and rs892145 depend on the heterozygous genotype, and such associations are always difficult to interpret [14–16]. It is noteworthy that the effect of the top SNPs, rs10888557 in *PGLYRP4*, and rs892145 in *PGLYRP2* are associated with decreased risk in the Goldman et al. report, while, in the Gorecki et al. study and the Luan et al. report (rs892145 only), the variants are more common in the patient groups, thus conferring an increased risk. Comparing the minor allele frequency (MAF) found in our Swedish cohort with the other studies, we find that the rs10888557 variant has unusually low frequencies in the Swedish population, while the MAF for rs892145 is comparable with the American control population. These numbers are also in accordance with reported MAFs in relevant populations (1000G, HapMap, and Northern Sweden) from publicly available databases (https://www.ncbi.nlm.nih.gov/snp). Our meta-analysis investigating the heterozygous association of rs892145 in the four cohorts was inconclusive because of the elevated heterogeneity. The heterogeneity was introduced by the opposite effect of the association observed in the American cohort, and by removing this cohort from the analysis in a post hoc analysis, we found an insignificant *I*^2^ heterogeneity score of 14.82 and an association under both fixed and random effects model; OR (random) = 1.32, *P* value 6.421*e* − 05. Importantly, it is likely that lifestyle factors would impact findings concerning the microbiota as well as the proteins involved in its regulation. In complex genetic disorders such as PD, the combination of genetic, lifestyle, and environmental factors confer the risk of the disorder. Lifestyle factors can vary greatly in different parts of the world. As we have no means of correcting for these in the present analysis, this may be considered a confounding factor when comparing data from different cohorts.

In our analyses stratified by sex, we discovered that the MAFs varied between males and females for rs892145 in *PGLYRP2*, and with opposite directions of effect. The minor allele of rs892145 conferred an increased risk of PD in men and a decreased risk of PD in women. However, these data do not hold for correction for multiple testing. There was also a trend for decreased gene expression of *PGLYRP2* in females. Luan et al. published sex-stratified allele counts, but they were not comparable to our data, as male and female patients displayed highly similar MAF [16]. The male-to-female ratio for Swedish PD patients is around 1.5 : 1 (1.7 : 1 in our study) [21]. In the Chinese cohort used for our meta-analysis, authors reported a ratio of 1.2 : 1; similarly, the sex distribution has been reported to be less pronounced in Asian PD populations as compared to European populations [16, 22]. Therefore, these data need to be verified in an independent European cohort before drawing conclusions.

One limitation of our study is the sample size, particularly for the gene expression analysis. With our sample size, the statistical power of our analysis permits the detection of genetic associations with an OR in the range of <0.7 or >1.5 which is slightly above our reported ORs. Consequently, replication studies are warranted, and our results should be interpreted with caution.

The rs892145 variant has previously been associated with irritable bowel syndrome (IBS) in an American cohort, with different MAFs for males and females [23]. Nonmotor symptoms vary slightly between male and female PD patients. Gastrointestinal problems occur with both sexes, but while men are at higher risk of sialorrhea (dribbling of saliva), women more often report constipation [24]. However, gastrointestinal problems are more common in females in the general population, and in a study correcting for the prevalence in a matched control group, male patients had a generally higher risk of gastrointestinal disturbance than female patients [25]. Moreover, Pglyrp2 knockout (KO) mice have been shown to display a sex-specific motor and behavioral phenotype at an older age with improved motor coordination and increased anxiety-like behavior in female mice [26]. Previous results from our research suggest that the expression of Pglyrp2 in mice is both sex- and age-dependent with young female mice having the most elevated expression levels [27]. Thus, several studies support the hypothesis of a sex-dependent effect of PGLYRP2 dysregulation. Animal studies further suggest that there is also an age-dependent effect, with a potentially beneficial motor function phenotype in older women, but this remains to be investigated in humans.

PGLYRP2 is the only mammal PGLYRP with amidase activity and has an anti-inflammatory effect in the gut [28, 29]. As a consequence, alterations in PGLYRP2 activity may result in dysbiosis and thereby potentially affect PD. In allowing a more proinflammatory environment in the gut, PGLYRP2 dysfunction could lead to the development or acceleration of *α*-synuclein pathology. Increased expression of *α*-synuclein has been found in Pglyrp2 KO mice [26]. Another potential pathway is a direct effect on the brain. Studies have shown that PGLYRP2 affects brain development [27], and peptidoglycans have been linked to neurological inflammation and multiple sclerosis (MS) in the past [30]. Future studies will hopefully shed light on whether microbiota or related signaling pathways are involved in PD pathophysiology, or whether the observed changes in the microbiota of PD patients are a consequence of other pathological events.

## 5. Conclusion

In conclusion, *PGLYRP* genes are interesting as potential players in PD pathophysiology because of their link to gut microbiota. Our data do not indicate that genetic variants in *PGLYRP*s directly affect the risk of PD but suggest a possible link with sex differences in PD. PD is more common in men than in women, and in our data, the minor allele of rs892145 was overrepresented in male patients and underrepresented in female patients. Further studies in independent cohorts are required to investigate whether rs892145 can be correlated to the elevated incidence of PD in the male population in Europe and whether this variant is involved in PD pathology.

## Figures and Tables

**Table 1:** Demographic information of the Swedish cohort.

	Male (%)	Average age (years)	Average age of onset years (range)	Family history of PD (%)
PD (*n* = 508)	62.8	68 (37–90)	60.4 (29–89)	28.1^a^
Control (*n* = 585)	45.8^b^	71.9^c^ (24–90)	NA	NA

PD: Parkinson's disease, *n*: number of individuals, NA: not applicable, ^a^information available for 320 patients, family history was defined as having one or more first-, second-, or third-degree relatives diagnosed with PD, ^b^information available for 582 control individuals, and ^c^information available for 394 control individuals.

**Table 2:** Results from the genetic analysis.

Gene	SNP	Allele	Control % (*n*)	PD % (*n*)	OR (95% CI)	*P* value
*PGLYRP2*	rs892145	A	63.2 (733)	62.6 (617)	1.01 (0.85–1.21)	0.96
		T	36.8 (427)	37.4 (369)		
	Female strata	A	61.2 (382)	67.9 (250)	0.76 (0.58–0.99)	0.04
		T	38.8 (242)	32.1 (118)		
	Male strata	A	65.2 (347)	59.4 (367)	1.29 (1.01–1.64)	0.04
		T	34.7 (185)	40.7 (251)		
	rs959117	T	51.4 (588)	50.2 (494)	1.05 (0.88–1.24)	0.61
		C	48.6 (556)	49.7 (490)		

*PGLYRP4*	rs10888557	G	90.9 (918)	91.2 (598)	1.01 (0.71–1.43)	0.96
		C	9.1 (92)	8.8 (58)		

SNP: single nucleotide polymorphism, *n*: number of alleles, PD: Parkinson's disease, OR: odds ratio, CI: confidence interval. Data were analyzed with logistic regression with sex as a covariate for the entire cohort and stratified by sex as indicated for rs892145.

## Data Availability

The raw data supporting the conclusions of this article will be made available by the authors, without undue reservation.
